# Diffusion Tensor Imaging-Based Studies at the Group-Level Applied to Animal Models of Neurodegenerative Diseases

**DOI:** 10.3389/fnins.2020.00734

**Published:** 2020-08-31

**Authors:** Hans-Peter Müller, Francesco Roselli, Volker Rasche, Jan Kassubek

**Affiliations:** ^1^Department of Neurology, University of Ulm, Ulm, Germany; ^2^German Center for Neurodegenerative Diseases (DZNE), Ulm, Germany; ^3^Core Facility Small Animal MRI, University of Ulm, Ulm, Germany

**Keywords:** DTI, magnetic resonance imaging, translational, *in vivo* animal model, neurodegeneration, group studies

## Abstract

The understanding of human and non-human microstructural brain alterations in the course of neurodegenerative diseases has substantially improved by the non-invasive magnetic resonance imaging (MRI) technique of diffusion tensor imaging (DTI). Animal models (including disease or knockout models) allow for a variety of experimental manipulations, which are not applicable to humans. Thus, the DTI approach provides a promising tool for cross-species cross-sectional and longitudinal investigations of the neurobiological targets and mechanisms of neurodegeneration. This overview with a systematic review focuses on the principles of DTI analysis as used in studies at the group level in living preclinical models of neurodegeneration. The translational aspect from *in-vivo* animal models toward (clinical) applications in humans is covered as well as the DTI-based research of the non-human brains' microstructure, the methodological aspects in data processing and analysis, and data interpretation at different abstraction levels. The aim of integrating DTI in multiparametric or multimodal imaging protocols will allow the interrogation of DTI data in terms of directional flow of information and may identify the microstructural underpinnings of neurodegeneration-related patterns.

## Introduction

In this systematic review, principles of diffusion tensor imaging (DTI) analysis at the group level with the special focus on applications to animal models of neurodegeneration are summarized. Methodological aspects are addressed covering experimental design and DTI data acquisition as well as data analysis at the group level. The emphasis will be on the concept of translational imaging from *in-vivo* animal models of neurodegeneration to (clinical) applications in humans that may sometime form the basics for novel therapeutic approaches. The continuous and compulsive research is addressed in studying the patterns underlying cellular and molecular relations in living animals since there are at present no sufficient *in-vitro* or *in-silico* models that can serve as alternatives to the use of *in-vivo* animal models (Bennett and Ringach, 2016). Thus, insights into the spectrum of DTI-based neuroimaging data analysis is provided and interpretations at different abstraction levels in that context are summarized.

### Neuroimaging of Neurodegenerative Diseases in Humans and Preclinical Models

Structural and microstructural neuroimaging findings especially by magnetic resonance imaging (MRI) have improved the longstanding notions regarding the pathophysiology of neurodegenerative diseases (Frisoni et al., 2010; Chiò et al., 2014; Politis, 2014; Blamire, 2018). The cellular mechanisms underlying the stereotypical progression of pathology in specific neurodegenerative diseases are not completely understood; however, there is increasing indication that misfolded protein aggregates can spread by a self-perpetuating neuron-to-neuron transmission (Braak et al., 2013; Jucker and Walker, 2013, 2018; Goveas et al., 2015). Neuroimaging techniques can identify specific lesion patterns and explain how these disorders spread across brain networks (Agosta et al., 2015). However, most neuroimaging studies have drawbacks, such as limited sample sizes in orphan diseases or insufficient clinical characterization of patients, absence of adequate controls, and scarcity of longitudinal assessments.

This review concentrates on DTI, as a subtechnique of diffusion-weighted imaging, in the application to animal models of neurodegenerative diseases. A special focus are animal models of dementia like Alzheimer's disease (AD), motor neuron disorders such as amyotrophic lateral sclerosis (ALS), and Parkinson's disease (PD), although the neuropathological disease-spreading concept in humans in predefined patterns related to the forming of pathogenic assemblies of disease-specific proteins (“prion-like paradigm”) could not be demonstrated in an animal model yet.

Animal models of adult-onset neurodegenerative diseases have helped to understand the molecular pathogenesis of these diseases. Despite all limitations, the understanding of these disorders and the improvement of mechanistically designed therapeutics can still profit from these animal models and from the generation of animal models that more exactly recapitulate human disease (Dawson et al., 2018). However, the characterization of any new model is crucial and remains a bottleneck; efforts have to be performed to comprehensively catalog the phenotypes associated with each model, including studies such as *in-vivo* imaging (Dawson et al., 2018).

### DTI Mapping of White Matter

White matter tracts of the central nervous system consist mainly of densely packed axons and various types of neuroglia. The axonal membrane and myelin layers are the predominant biological features that restrict the water diffusion perpendicular to the fiber orientation. This, leads to an anisotropic water diffusion in brain white matter. Additionally, myelin sheaths around the axons contribute to the anisotropy of diffusion for intra- as well as for extracellular water (Mori and van Zijl, 2002; Garin-Muga and Borro, 2014).

The quantitative description of this anisotropy is measured by DTI, imaging the local microstructural characteristics of water diffusion. The signal intensity in each recording voxel is attenuated depending on the amplitude and the direction of the diffusion-encoding gradients as well as on the local microstructure in which the water molecules diffuse (Basser et al., 1994). In the presence of anisotropy in white matter, diffusion properties can be described in first approximation by a tensor (Mattiello et al., 1994). The anisotropy of the diffusion processes is related to the presence and orientation of fiber tracts in white matter and is therefore influenced by its micro- and macrostructural features. On a macroscopic scale, the intra-voxel coherence in the orientation of all white matter tracts in an imaging voxel influences its degree of anisotropy, whereas the microstructural features, mainly the intraaxonal organization besides the density of fiber and cell packing, degree of myelination, and individual fiber diameter, influence diffusion anisotropy (Pierpaoli and Basser, 1996; Duan et al., 2015).

In DTI recordings of the human brain, the voxel dimensions are in the order of millimeters. Thus, a voxel always contains the averaged information of diffusion covering a high number of axons as well as the surrounding water molecules. In spite of this multidirectional environment, DTI recordings are sensitive to the orientation of the largest principal axis, which aligns to the predominant axonal direction, that is, the axonal contribution dominates the recorded signal (Mori and van Zijl, 2002; Brandstack et al., 2016).

DTI techniques provide basically several types of information about the property of water diffusion: first, the orientation-independent extent of diffusion anisotropy (Pierpaoli and Basser, 1996) and second, the predominant direction of water diffusion in image voxels, that is, the diffusion orientation (Pajevic and Pierpaoli, 1999; Marrale et al., 2016). The diffusion tensor model allows for the calculation of multiple parameters; out of these, the fractional anisotropy (FA) is the most commonly used parameter to measure directional dependence of water diffusion that way parameterizing the shape of the tensor and providing a normalized value to the degree of anisotropy (Sampaio-Baptista and Johansen-Berg, 2017).

### DTI in the Animal Brain—Translational Imaging

DTI has become an important tool to study the anatomy of animal brains *in vivo*, for example, the mouse brain (Aggarwal et al., 2010; Harsan et al., 2010; Nouls et al., 2018), the rat brain (Gyengesi et al., 2014; Figini et al., 2015), the canine brain (Wu et al., 2011), or the primate brain (Feng et al., 2017; Risser et al., 2019). The non-invasive nature of MRI/DTI enables longitudinal studies of transgenic disease models (Haber et al., 2017; Petrella et al., 2018). Ultra-high fields at 11.7 T (Müller et al., 2012a) or 16.4 T (Brennan et al., 2013) and dedicated resonators [cryogenic cooled resonator (CCR)] allow the recording of high-resolution DTI datasets with in-plane resolutions down to 100 × 100 μm for mouse models (or small rodents) with an axial slice thickness down to 200 μm (Müller et al., 2012a), while the development of fast DTI protocols has led to reduced acquisition times until about 30 min, enabling the logistics for the monitoring of larger cohorts (Müller et al., 2013).

In DTI, the diffusion in each voxel is assumed to be represented by a single tensor. In human brain imaging, where typical voxel sizes can be easily as high as 2 × 2 × 2 mm^3^, this assumption fails in regions where multiple fiber bundles of various spatial orientations may be packed together in a single voxel (“crossing fibers”). In the same line, DTI does not account for multiple tissue types within a voxel, and a number of different white matter microstructural features (including the cellular membranes of glial cells and the axon diameter and density) contribute to the obtained DTI indices (Sampaio-Baptista et al., 2019). The ratio of the microstructural feature size to voxel size is improved in small animal imaging where voxel sizes for mice and small rodents, typically in the order of 100 × 100 × 200 μm^3^ *in vivo* and even much higher spatial resolution up to isotropic some 10 μm *ex vivo*, are much closer to the fiber bundle diameters (Aggarwal et al., 2010; Kumar et al., 2014).

## Methods

### Search Strategy and Study Selection

**Figure 1:**
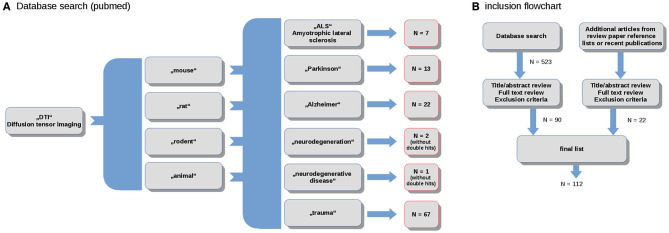
**(A)** Selection diagram depicting the number of studies included for the spectrum of studied neurodegenerative diseases; and **(B)** PRISMA flowchart.

The literature review and study inclusion process were conducted in accordance with the PRISMA guidelines (Panic et al., 2013). In a systematic search conducted in April 2020, data were collected from the online library PubMed (https://www.ncbi.nlm.nih.gov/pubmed/). The search keywords were the combination of the terms (“DTI”) and (“mouse,” “rat,” “rodent,” “animal”) and (“ALS,” “Parkinson,” “Alzheimer,” “neurodegeneration,” “neurodegenerative disease,” “trauma”). In total, this search yielded more than 500 results. These studies were probed for original research and English language in peer-reviewed journals. Studies with models that do not refer to neurodegenerative diseases were excluded. The references were studied for further candidates. In total, 114 studies were included in the systematic review (Figure 1).

### DTI Data Acquisition: Scanners and Coils

The major challenges in *in-vivo* DTI is the long acquisition time required to acquire the multi-directional diffusion data with sufficient signal-to-noise ratio (SNR) at the required spatial resolution. While scanning time for *ex-vivo* studies is in principle without direct limitation, *in-vivo* experiments require careful consideration of animal welfare. Since animals are usually anesthetized, longer scan times than in human application are possible, and adult animals can in principle be scanned for several hours, yielding high spatial resolution and sufficient SNR for subsequent DTI analysis with species-optimized MR scanning protocols and even systems (Oguz et al., 2012; Rumple et al., 2013; Zhang et al., 2013). Prolonged scan times might rise concerns regarding motion artifacts (Oguz et al., 2014; Zhang X. et al., 2016) despite the use of dedicated holders additionally to the general anesthesia (Herrmann et al., 2012; Müller et al., 2013; Zhang et al., 2015); however, drift or misalignment can readily be corrected during post-processing. Further, reduction of scan times is of general interest to enable large cohort studies.

**Figure 2:**
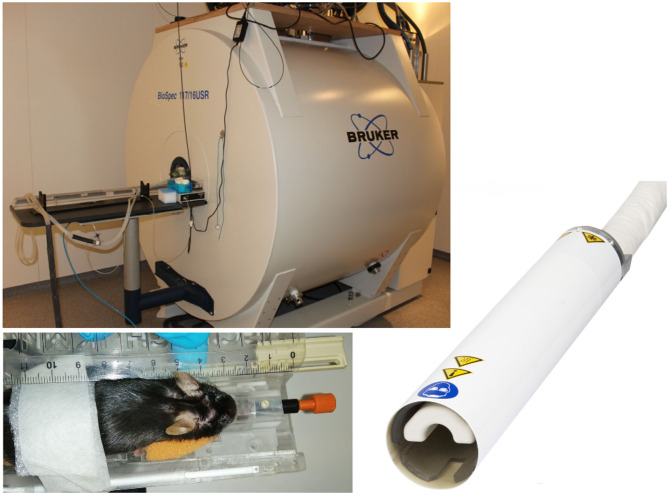
Typical equipment for scanning of mouse cohorts. 11.7 T tomograph (Biospec 117/16, Bruker, Ettlingen, Germany), two-element transmit/receive ^1^H mouse cryogenic surface coil (Cryo-Probe, Bruker BioSpin); the animals are placed in a stereotaxic head support (Bruker Biospin, Ettlingen, Germany) to immobilize the head. Data acquisition is performed under isoflurane anesthesia.

Where non-human primate and rat studies have even been performed on clinical systems (Mayer et al., 2007; Zhang et al., 2013; Zhang R. Z. et al., 2016), recent advances in high-field MR imaging offer improved SNR and resolution. Especially for rodent imaging, typically specialized coils and high-field scanners (up to 17.6 T) are applied. DTI studies of the rodent brain have been reported using dedicated small animal systems from 4.7 T up to 17.6 T (Nair et al., 2005; Duong, 2010; Harsan et al., 2010; Lodygensky et al., 2010; Gatto et al., 2018a). For further improvement of SNR, the use of dedicated application-specific receive coils ranging from simple single-loop surface coils to complex phase array and micro-imaging coils (Zhao et al., 2008) has been introduced. In the field of rodent imaging, most promising are CCRs, which have demonstrated significant increase of the effective SNR (Ratering et al., 2008) at 9.4 T and are supposed to provide an SNR gain of at least a factor of 2 at 11.7 T systems (Figure 2). In mice, the use of CCR at 11.7 T has enabled the reduction of the DTI acquisition times to ~30 min, which is considered feasible for cohort studies (Müller et al., 2012a).

### DTI Data Acquisition: Pulse Sequences

DTI relies on fast diffusion encoding imaging sequences. Although advanced acquisition techniques including spiral (Frank et al., 2010) and gradient and spin echo GRASE (Aggarwal et al., 2010) imaging techniques have been introduced to *in-vivo* DTI, independent of the field strength and animal model, most reported studies still rely on conventional single-shot or segmented echo planar imaging. Ideally, isotropic 3D imaging techniques are used to provide high-fidelity DTI data. However, with acquisition times in the several hour range (Cai et al., 2011; Wu et al., 2013), application to cohort studies is limited and conventional 2D multi-slice techniques are still broadly used. As the current standard for murine cohort studies (and other rodents), multi-slice echo planar imaging acquisitions with echo times between 50 and 100 ms and repetition times between 6,000 and 15,000 ms are frequently used. Spatial in-plane resolution and slice thickness is adapted to the size of the animal model with in-plane spatial resolution ranging from 100 × 100 μm^2^ (mice) to 650 × 650 μm^2^ (chimpanzee). For volume of interest coverage, typically 50–100 axial slices with 200 μm (mice) to 1,000 μm (chimpanzee) slice thickness are acquired. To ensure sufficient diffusion tensor fidelity, diffusion weighted (typically *b* = 1,000 s/mm^2^) images are acquired with 30 and more different encoding (gradient) directions plus one to five unweighted (*b* = 0 s/mm^2^) images. There is no general rule for the optimum sampling scheme in DTI; the performance of sampling schemes that use low numbers of sampling orientations and less efficient schemes with larger numbers of sampling orientations and the scenarios in which each type of scheme should be used (Jones, 2004) are still under discussion. Scanning times for sufficient spatial resolution, SNR, and DTI quality have been reported in the hour range. Even though still limited by SNR constraints, parallel imaging and compressed sensing techniques (Shi et al., 2015) have been introduced to further reduce scan times and high spatial resolution DTI in mice was shown to be feasible within 30-min scan times (Müller et al., 2012a), thus enabling application to large cohort studies.

### Atlases

Animal MRI/DTI analysis requires atlases in analogy to the standardized coordinate frames for humans such as the Montreal Neurological Institute atlas (Brett et al., 2002). High-resolution images are used for the identification of rodent brain regions by human experts to delineate regions of interest (ROI) or tracts of interest (TOI). Prominently used atlases are summarized in the following (Oguz et al., 2012).

Mouse atlases:

- Waxholm atlas (Jiang and Johnson, 2009) includes four adult male C57 mice and includes T1, T2, T2^*^, and DTI images at high resolution.
- Developmental Mouse Atlas (Zhang et al., 2003; Aggarwal et al., 2009; Chuang et al., 2011) is a series of atlases of developing mouse embryos, including FA, T2w, and tensor maps collected from mouse fetuses at embryonic days.
- The mouse brain in stereotaxic coordinates (Paxinos and Franklin, 2007) providing both accurate stereotaxic coordinates for laboratory use, and detailed delineations and indexing of structures for reference.
- The online Allen Reference Brain Atlas (available at https://mouse.brain-map.org/static/atlas) including sagittal and coronal implementation of the Allen Mouse Common Coordinate Framework.

Rat atlases:

- A DTI-based atlas of the rat brain (Rumple et al., 2013)
- The rat brain in stereotaxic coordinates (Paxinos and Watson, 2013), providing both accurate stereotaxic coordinates for laboratory use, detailed delineations, and indexing of structures for reference.

Primate atlases:

- A digital 3D atlas of the marmoset brain based on multi-modal MRI (Liu C. et al., 2018).
- The marmoset brain in stereotaxic coordinates (Palazzi and Bordier, 2008).

### Data Processing

For DTI analysis many automated and manual tools are available. With respect to the large amount of data that are recorded during a DTI experiment, only tools should be considered that are necessary to address the endpoints of the specific study, that way maintaining statistical power and reducing analysis time. Regardless of the tools selected, there are typically a number of steps necessary to acquire data from DTI images (Oguz et al., 2012).

In analogy to human DTI analysis, some preprocessing steps are necessary on DTI data (Liu et al., 2010). These include eddy current corrections, rigid registration of individual diffusion weighted images to the baseline image to minimize motion effects, correction of intensity inhomogeneities, quality control, and elimination of possible corrupted gradient directions (Müller et al., 2012a; Oguz et al., 2012) as well as the stereotaxic normalization to a brain atlas coordinate frame (cf. 2.3).

**Figure 3:**
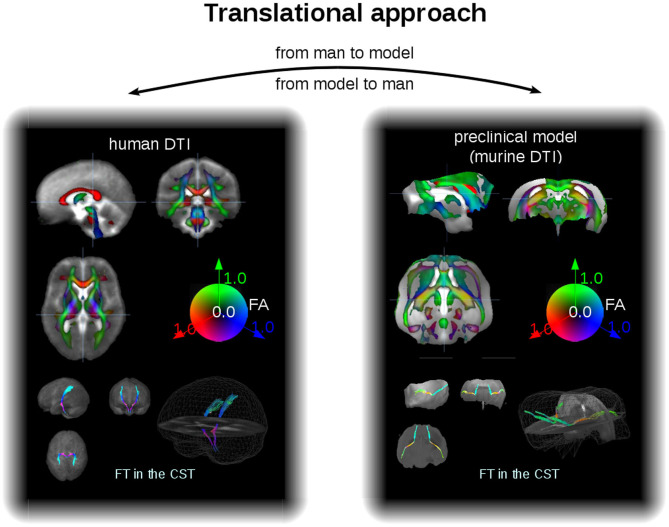
Translational approach. In a different scale, DTI data of man and mice can be analyzed within the same software environment, that is, with the same analysis cascade. Examples for FA maps in coronar, sagittal, and axial orientation (upper panel) and projectional views of FT examples (lower panel) for human (left) and murine (right) group averaged data sets. The cerebellum of the mouse brains has been masked out. CST, corticospinal tracts.

As an example for the data analysis cascade, data processing is described as performed with the *Tensor Imaging and Fiber Tracking* software package, which has been successfully applied both to human DTI group studies in neurodegenerative diseases (Kassubek and Müller, 2016) and to data of preclinical models of neurodegeneration (Müller et al., 2012a). Thus, with a rescaling, the same software analysis cascade can be applied to analysis of human and murine DTI data, that way consolidating the translational aspect both “from man to preclinical model” and vice versa “from preclinical model to man” (Figure 3). For animal as well as for human DTI studies, a slice thickness to in-plane resolution ratio between 1.0 and 1.5 is considered to be a good choice. For animal studies, the recorded brain grid could be adjusted to be in the same order as in human DTI studies, since the transformation to an iso-grid of 50 μm for mice (and corresponding values for other animals) corresponds to an iso-grid of 1 mm in human studies. After transformation of the recorded data into an iso-grid by nearest neighbor interpolation, spatial normalization to a stereotaxic standard space is performed using study-specific b0- and FA-templates (Müller et al., 2012a). Optimum normalization is obtained by an iterative process using scanner- and sequence specific b0- and FA-templates according to landmarks of a stereotaxic animal atlas (see section DTI Data Acquisition: Pulse Sequences).

**Figure 4:**
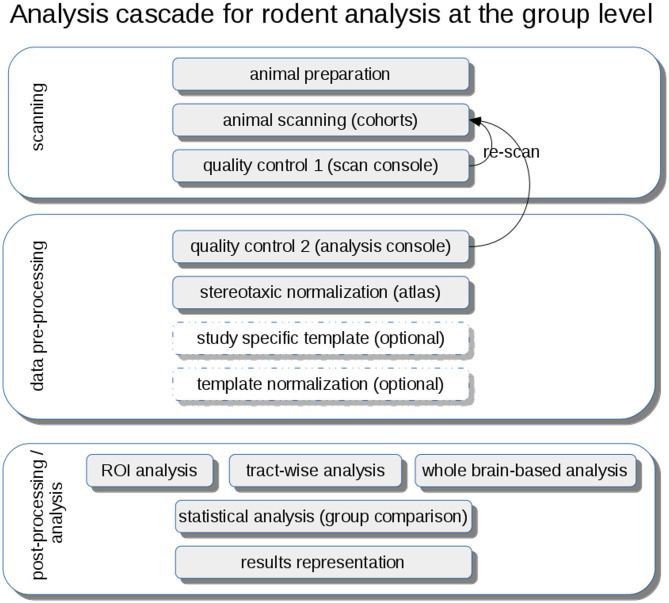
Analysis cascade for rodent analysis at the group level. After animal preparation and scanning, quality control could be performed first at the scan console and second during the pre-processing at the analysis console with the option of re-scanning of low-quality data sets. A stereotaxic normalization to atlas coordinates can be refined by the creation of study-specific templates and a subsequent template normalization. Analysis at the group level can be performed by hypothesis-guided ROI analysis or tractwise analysis or by unbiased whole brain-based analysis, each followed by a statistical analysis extracting and representing significant results using a pre-defined significance level.

DTI metrics maps (FA, axial diffusivity—AxD, radial diffusivity—RD, mean diffusivity—MD) are calculated from these stereotaxically normalized data sets and are in a following step smoothed with a Gaussian filter with a size of about two to three times the recording voxel size, that way providing a good balance between sensitivity and specificity. The axonal damage and myelin degradation is mirrored by DTI metrics; differences at the group level to assess microstructural alterations by statistical analysis of DTI metrics can be performed by various approaches (Müller and Kassubek, 2018): (1) unbiased voxelwise comparison by whole brain-based spatial statistics (WBSS) (Müller et al., 2012b) or tractwise comparison by tract-based spatial statistics (TBSS) (Smith et al., 2006) or (2) hypothesis-guided tract-based quantification by analyzing DTI metrics in tract systems by TOI-based tractwise FA statistics (TFAS) (Müller et al., 2007) (Figure 4).

### Whole-Brain-Based Spatial Statistics

Unbiased voxelwise comparison of cohort brains can be performed for cross-sectional comparisons of mutant animals vs. *wt* animals at baseline or at follow-up, using WBSS (Müller et al., 2012a). Statistical comparisons of DTI metrics maps for mutant vs. *wt* are performed voxelwise by means of statistical testing [a good choice is an FA threshold of 0.2 (Kunimatsu et al., 2004) to concentrate the analysis on white matter], followed by correction for multiple comparisons (e.g., with the false-discovery-rate algorithm at *p* < 0.05 Genovese et al., 2002) and further reduction of the alpha error by a spatial correction algorithms in the size range of the smoothing kernel, leading to a minimum size of alteration clusters.

Voxelwise comparison (e.g., by WBSS) of longitudinal DTI map differences could be performed by calculating voxelwise differences between DM maps of baseline and follow-up scans for mutant animals and *wt* animals; differences have then to be linearly normalized to an identical time-interval prior to statistical comparison. In analogy to cross-sectional WBSS, statistical comparisons of longitudinal DTI map differences are performed voxelwise by means of statistical testing, and results have to be corrected for multiple comparisons and also by clustering.

### Whole-Brain Connectivity Analysis

A connectome refers to a comprehensive description of neuronal connections, for example, the wiring diagram of the entire brain. Given the enormous range of connectivity in the mammalian brain, such descriptions on a macro- or mesoscale range (Oh et al., 2014) can be inferred from imaging white matter fiber tracts through DTI in the living brain (Nouls et al., 2018). DTI-based connectomics have already been applied to mouse models of genetic risk factors for late onset AD for identifying vulnerable brain networks (Badea et al., 2019). Whole mouse brain structural connectomics (Shibata et al., 2015; Allan Johnson et al., 2019) have already been verified by neuron tracing data (Chen et al., 2015; Sinke et al., 2018; Wang et al., 2018).

### Region-of-Interest Analyses

A hypothesis-guided approach is performed when using ROIs that are placed in defined anatomical regions and comparing the average DTI metrics values, that is, rotational invariant parameters of the diffusion tensor, within the respective ROI for the cohorts (e.g., Harsan et al., 2010; Müller et al., 2019) for quantitative comparisons of in-between ROIs or to show differences between various white matter regions. ROI analysis could be extensively performed by placing an arbitrary number of ROIs (e.g., Irie et al., 2018) with variable extension. The advantage of ROI analysis is that (in case of accurate anatomical placement) it can also be performed without any prior stereotaxical normalization; in this case, manual ROI identification can be supported by confocal microscopic image (Irie et al., 2018).

### Tract-Based Analysis

In order to identify fiber structures in an atlas-based coordinate frame, different technical approaches can be applied. One technique is TBSS (Smith et al., 2006), a fully automated method to perform whole-brain tract DTI analyses by calculating a skeleton of fibers by projection onto an alignment-invariant tract representation. TBSS has been shown to be applicable not only to human DTI studies, but also to different animal models (Sierra et al., 2011). An additional more tract specific technique is the TOI-based approach for which an averaged data set is created from all contributing data sets after normalization to a common coordinate frame preserving all tensor-related information (Alexander et al., 2001; Field et al., 2005). Then, fiber tracking (FT) techniques could be applied, e.g., deterministic streamline tracking in order to obtain defined tract structures. These tract structures are used in order to obtain a quantitative access to the tractography results (TFAS—Müller et al., 2007) by comparing the average DTI metrics values within the respective TOI for the cohorts. This TOI analysis approach can be used for a hypothesis-guided analysis of FT bundles.

### Advantages and Drawbacks of the Different Analysis Techniques

Unbiased comparison of cohort brains can be performed by WBSS or connectome/whole brain connectivity analysis either cross-sectionally or longitudinally. The drawback of these approaches is that they are less specific, and one has to deal with statistical correction algorithms, such as corrections for multiple comparisons and spatial cluster corrections. WBSS requires no fiber tracking algorithms, thus is not depending on all the accuracy challenges, which FT/connectome analysis has to deal with.

Hypothesis-guided techniques, that is, ROI analysis or tract-based analysis, have the advantage to specifically focus on certain brain regions also at the individual level with or without any prior stereotaxical normalization. However, the comparison of the results to reference regions is mandatory to obtain an impression about the validity of the results.

In general, FT-based techniques have the advantage to analyze also long-range structural connections, whereas ROI techniques are restricted to certain brain areas. However, as FT techniques are much more sophisticated and probably contain a higher sensitivity to widespread brain alterations, the possibility of misinterpretation and unspecific erroneous results is increased.

## Applications to Models of Neurodegenerative Diseases

### Methodological DTI Studies in Cohorts of Preclinical Models in General

DTI in mouse cohorts has successfully been applied to image microstructure organization with a resolution down to 50 μm (Aggarwal et al., 2010; Guilfoyle et al., 2011). The analyses have been performed by ROI analysis or fiber tracking techniques (Harsan et al., 2010; Müller et al., 2012a). As one example, the translational role of DTI in developmental pathologies has been extensively described (Oguz et al., 2012). Special focus has been put on the identification of rodent olfactory bulb structures with micro-DTI (Zhao et al., 2008) or DTI tractography analysis of infralimbic and prelimbic connectivity using high-throughput MRI (Gutman et al., 2012). In the following chapters, a summary for AD, PD, and ALS is provided; single studies are concerned with, for example, a risk-related biomarker in animal model of glaucoma (Hayashi et al., 2013) or neurodegeneration in Niemann-Pick type C mice (Totenhagen et al., 2012). A comprehensive review of DTI in preclinical studies of Huntington's disease has recently been published by Gatto and Weissmann (2019) so that Huntington's disease was not included in our review.

### Cohort DTI Studies in Animal Models of Amyotrophic Lateral Sclerosis (ALS)

Cohort DTI studies have been performed for the SOD1 mouse model of ALS for the spinal cord (Kim et al., 2011; Underwood et al., 2011; Marcuzzo et al., 2017), detecting longitudinal white matter degeneration alongside histology and electron microscopy. DTI of ALS brains of SOD1-mice at 9.4 T and 16.7 T, respectively, have shown a presymptomatic decrease in axonal organization by FA and neurite content by Intracellular Volume Fraction across the spinal cord, corpus callosum, hippocampus, and cortex; the combination of DTI, neurite orientation dispersion, and density imaging (NODDI), and diffusion kurtosis imaging (DKI) (Marrale et al., 2016) models have proved to provide an assessment of the early microstructural changes in the ALS brain (Gatto et al., 2018b,c, 2019). Longitudinal DTI in the *TDP-43*^*G*298*S*^ ALS mouse model at the cohort level revealed cortical and callosal microstructure alterations (Müller et al., 2019); in this study, longitudinal DTI scans at 11.7 T of baseline and follow-up scans with an interval of several months were investigated by voxelwise comparison as well as by tractwise analysis, while histological investigations complemented the *in-vivo* results.

### Cohort DTI Studies in Animal Models of Alzheimer's Disease (AD)

Seminal work on the application of DTI to AD mouse models has been obtained for the first time more than 15 years ago (Sun et al., 2005; Shepherd et al., 2006), with the identification of age-dependent white matter disturbances in mice overexpressing beta-amyloid precursor protein (APP) under control of the platelet-derived growth factor promoter (PDAPP mice) (Song et al., 2004). This work was followed up by a detailed cohort study of APPsw transgenic mouse (Tg2576), which revealed abnormal DTI metrics related to axonal damage (both in gray and white matter) in mice of 12 months of age (or older) and abnormal DTI metrics related to myelin damage at 16 and 18 months of age (Song et al., 2005). However, these findings were not confirmed in *ex-vivo* DTI measurements, which revealed no loss in white matter integrity (Harms et al., 2006), raising questions about the source of the signal actually lost upon formalin fixation. More recently, DTI abnormalities have been confirmed in multiple white matter tracts as well as in the hippocampus in different mouse models for AD (Kerbler et al., 2013; Snow et al., 2017) and imaged *in vivo* at different field strengths, for example, 7 T (Whittaker et al., 2018) or 11.7 T (Zerbi et al., 2013). Further investigation of the APP model has confirmed abnormalities in DTI metrics both in gray and in white matter (Bitner et al., 2012; Qin et al., 2013; Shu et al., 2013; Shen et al., 2018; Liu L. et al., 2020). Gray and white matter degeneration was detected by DTI in an unbiased approach in cohort studies of APP transgenic mice (Müller et al., 2013) and 3× Tg-AD model mice (Manno et al., 2019) or after Aβ injections (Sun et al., 2014; Nishioka et al., 2019). *Ex-vivo* DTI was used to identify vulnerable brain networks in mouse models for late onset AD (Hara et al., 2017; Badea et al., 2019).

Histological confirmation demonstrated that such abnormalities corresponded to cortical and hippocampal neuronal loss, dendritic dystrophy and plaque accumulation, perivascular space dilation, and myelin damage (Qin et al., 2013). However, study of the triple-transgenic AD mouse model carrying mutations in APP, PS1, and the P301L mutation in Tau genes could not detect abnormalities in DTI metrics (imaged at 7T) despite histological detection of plaques and tangles-like lesions (Kastyak-Ibrahim et al., 2013). More recently, DTI imaging has been used to study murine models of tauopathies, namely the rTg4510 carrying the Tau(P301L) transgene; FA was found to be significantly decreased in the corpus callosum of these mice when imaged at 8.5 months of age at 9.4 T (Wells et al., 2015). In this model, DTI was sensitive enough to detect changes, histologically confirmed, in several areas of corpus callosum as early as 2.5 months after birth (Sahara et al., 2014). In a similar model, DTI proved sensitive enough to detect the regeneration of myelin when the expression of mutant Tau was suppressed (Holmes et al., 2016).

Taken together, the majority of the DTI studies in AD murine models or rat models (Anckaerts et al., 2019) appear to detect abnormalities in FA and other metrics in white matter and, less consistently, in gray matter. Thus, DTI imaging appears to be a possible non-invasive approach to assess cortical and white matter integrity in AD mouse models.

### Cohort DTI Studies in Animal Models of Parkinson's Disease (PD)

Animal models of PD might address various aspects of the disease and its management, that is, the examination of pathogenetic mechanisms not only in the nigrostriatal system (van Camp et al., 2009; Zhang et al., 2017) but also in other brain regions and outside the brain, the investigation of the compensatory mechanisms under dopamine deficiency, the search of biological markers for presymptomatic parkinsonism, and finally the development of preventive therapy (Ugrumov et al., 2011). Early quantitative DTI studies reported decreased FA in the substantia nigra, indicating dopaminergic nigral degeneration in 1-methyl-4-phenyl-1,2,3,6-tetrahydropyridine (MPTP)-treated animals (Boska et al., 2007). Current technical studies investigated the value of DTI markers in the application to different PD models, such as MPTP and 3,4-methylene-dioxy-methamphetamine (MDMA) lesions, respectively, in the non-human primate: their different patterns could be demonstrated since MPTP-induced lesions were associated with MD increases of within the caudate and the anterior cingulate cortex, whereas MDMA-induced lesions were associated with FA increase within the caudate (Météreau et al., 2018). In another technical study, multiparametric MRI including DTI demonstrated the different characteristics of the rotenone and the 6-hydroxydopamine (6-OHDA) model after substantia nigra injection in rats, since the FA value of the substantia nigra was remarkably lower at 6 weeks than at other time points in the rotenone group, while in the 6-OHDA group, the FA value was decreased at 1 week (Liu L. X. et al., 2018). In the characterization of another model called the MitoPark mouse, which is a genetic model of PD with a dopaminergic neuron-specific knock-out inactivating mitochondrial transcription factor A, DTI demonstrated reduced FA in the corpus callosum and the substantia nigra (Cong et al., 2016). Beyond the different models, preclinical parkinsonism has been characterized by DTI in different species. A voxel-based analysis of a 7 T DTI study in marmosets before and after MPTP administration revealed increased diffusivity in the bilateral nigrostriatal pathway, validated by *ex-vivo* microscopic tractographic images, which showed loss of fiber structures in the MPTP-treated brain (Hikishima et al., 2015) and a longitudinal combined morphometric and DTI study in cynomolgus monkeys revealed widespread and dynamic structural changes not only in the nigrostriatal pathway but also in other cortical, subcortical, and cerebellar areas (Jeong et al., 2018). Further DTI studies (within multimodal imaging protocols) demonstrated significantly altered diffusivity parameters (MD, AD, RD) in the nigrostriatal tract (in correlation with MPTP dose), but not in the substantia nigra or striatum, in the macaque nemestrina after application of MPTP (Shimony et al., 2018) and increased FA in the ipsi- and contralateral striatum after 3 weeks and increase of AxD and MD in the ipsilateral striatum in rats with 6-OHDA striatal lesions (Perlbarg et al., 2018). That way, DTI applications to various PD models could contribute to the mapping of the underlying pathophysiology, together with DKI as a non-Gaussian DTI approach, which demonstrated microstructural alterations when applied to transgenic mice overexpressing human wildtype a-synuclein under the murine Thy-1 promoter, that is, increases in the striatum and thalamus after 3 months and in the substantia nigra after 6 months (Khairnar et al., 2017). Beyond mere descriptive assessments of the disease models, DTI was used in multimodal neuroimaging studies as a measure for therapeutic evaluations. Here, in a first step, levodopa-induced dyskinesia in MPTP/MDMA-intoxicated monkeys, as a model of the “classical” treatment complication in humans, were assessed by PET imaging and MRI including DTI, and severity of levodopa-induced dyskinesia was correlated to MD decreases in the ventral striatum but were no more altered after lesion of serotonergic fibers and the second levodopa period, highlighting that DTI is complementary to PET to decipher pathophysiological mechanisms underlying treatment-associated complications (Beaudoin-Gobert et al., 2018). As an example, for the use in therapy monitoring in a prospective case–control animal study in rats with the 6-OHDA model, simple diffusion delivery (direct microinjection of the drug into the brain tissue) of rasagiline was assessed by DTI and T2^*^ mapping (Fang et al., 2018). The authors could show that FA values of the substantia nigra in the simple diffusion delivery treatment group were significantly higher at week 1 and lower at week 6 than that of the PD control group; given that higher T2^*^ parameters at week 6 showed the same pattern, the authors considered the combination to be more reliable than other traditional methods for evaluating the curative effect of PD drugs in animal models. In summary, DTI, as one element of multimodal neuroimaging, has demonstrated a growing importance over the recent years in preclinical PD models for the assessment of disease-related pathophysiology and started to be used as a marker of therapeutic interventions.

### DTI Study of Animal Models of Traumatic Brain Injury

In contrast to chronic neurodegenerative conditions, in which axonal and microstructural changes progress over months or years, in traumatic brain injury (TBI), damage to brain architecture takes place in seconds (primary damage). Nevertheless, further progressive alterations unfold over days or weeks (secondary damage). Furthermore, recent evidence shows that traumatic injury to the brain constitutes a powerful trigger of chronic neurodegenerative processes, in particular related to Tau protein (Chen et al., 2018; Stern et al., 2019). Thus, measuring initial and progressive damage to the brain upon TBI is the key to understand how the acute damage cause immediate neurological deficits and at the same time set in motion neurodegenerative cascades. We identified 23 papers exploiting DTI for the study of mouse models of TBI and 42 applying DTI to rat models.

Because of the sensitivity to axonal disruption, DTI has been extensively used to characterize diffuse axonal damage and long-range white matter tract injury in murine models to TBI. Initial evidence (Mac Donald et al., 2007a) showed that in controlled-cortical-injury TBI model, DTI detected alterations in the corpus callosum white matter underlying the trauma site with a stereotyped evolution of DTI signal over time (Mac Donald et al., 2007b). Notably, the alteration in radial anisotropy displayed the largest effect size and were directly correlated with histological measures of axonal damage (Li et al., 2013; Tu et al., 2016; also in rat models). FA maps have demonstrated substantial microstructural abnormalities also in case of experimental blast injury in mouse (Rubovitch et al., 2011; Hutchinson et al., 2018; Venkatasubramanian et al., 2020; Weiss et al., 2020) as well as in rat models (Budde et al., 2013; Begonia et al., 2014; Kamnaksh et al., 2014; Zhuo et al., 2015; Tang et al., 2017; Badea et al., 2018; Missault et al., 2019; Mohamed et al., 2020; San Martín Molina et al., 2020). DTI appeared sensitive enough to detect changes even in mild TBI models (Hylin et al., 2013; Takeuchi et al., 2013; Long et al., 2015; Li et al., 2016; Herrera et al., 2017; Kikinis et al., 2017; Wendel et al., 2018; Hoogenboom et al., 2019) and in distant locations within the brain up to 1 year after injury (Pischiutta et al., 2018). Interestingly, very-high field intensity DTI (14 T) has been successfully applied *ex vivo* for the analysis of closed TBI (CHIMERA model); this approach revealed abnormalities in FA and in AxD with a sensitivity and spatial resolution comparable to immunohistological approaches (Haber et al., 2017). Recently, DKI has been added to the MRI toolset for TBI investigation and has been able to detect changes in injured cortex in a CCI model of brain injury as soon as 5 h (Hansen et al., 2017; Soni et al., 2019). Although the DKI approach is in principle more sensitive to complex microstructural changes occurring upon trauma in mice and rats (Zhuo et al., 2015; Wang et al., 2018; Braeckman et al., 2019; Yu et al., 2019), its role is not established and remains object of investigation.

DTI has also been employed in the detection of axonal injury and microstructural changes following repeated mild TBI (which mimics the occurrence of head traumas in several sports). In this model, reduced values of axial diffusivity and mean diffusivity in the corpus callosum were found at 7 days post injury, in agreement with histological markers; notably, radial diffusivity was already altered in the cortical gray matter at 24 h but returned to baseline at the 7 days evaluation (Bennett et al., 2012). In a similar repeated-hit model, DTI has revealed disruption of axonal integrity in multiple white matter structures, irrespective of microhemorrhage detection (Robinson et al., 2017); substantial white matter damage was detected by DTI, together with histological approaches, in juvenile mice subject to repeated mild TBI (Yu et al., 2017; Lee et al., 2018). Similar alterations have been detected in rat models of repeated TBI (Calabrese et al., 2014; Singh et al., 2016; Wright et al., 2016; Qin et al., 2018; Kao et al., 2019) as well as in juvenile rat (Fidan et al., 2018; Wortman et al., 2018; Wright et al., 2018) or mouse (Rodriguez-Grande et al., 2018; Clément et al., 2020) cohorts subject to TBI. A few studies have applied *ex-vivo* DTI to obtain high-resolution maps of axonal disruption upon TBI, both in mouse (Weiss et al., 2020) and in rat (Donovan et al., 2014; Laitinen et al., 2015) models of brain trauma.

Finally, DTI-MRI has been used as read-out of treatment efficacy in rodents' models of TBI. In particular, DTI has revealed the beneficial effect on white matter integrity of activation of mitochondrial calcium fluxes (Parent et al., 2020), of autophagy modulators (Medina et al., 2017; Yin et al., 2018), estrogens (Kim et al., 2015, 2017), metamphetamine (Ding et al., 2013), erythropoietin (Robinson et al., 2016, 2018), tissue plasminogen activator (in mice; Xia et al., 2018), mGluR5 (in mice; Byrnes et al., 2012), and dietary modulations (Shultz et al., 2015; Schober et al., 2016; Tan et al., 2016). Notably, the detrimental effect of alcohol in TBI was also investigated by DTI (Kong et al., 2013). Taken together, these studies extensively highlight the sensitivity of DTI as readout of acute and subacute axonal damage in rodents TBI models.

Efforts are currently made to bring DTI to non-murine models of TBI. MRI has been successfully employed to study traumatic damage to piglets (sus scrofa domestia) in order to simulate pediatric TBI (Kinder et al., 2019) as well as to explore therapeutic strategies in adult minipigs (Georgoff et al., 2017; Nikolian et al., 2017). Recently, DTI has been implemented in minipigs (Simchick et al., 2019), revealing a remarkable similarity of the pattern of functional and structural connectivity between men and pigs and underscoring the translational value of porcine models. These experiments prove that the use of DTI-based MRI on swine models is possible but a full DTI study has not yet been performed. Initial studies of the application of DTI in non-human primates for the investigation of TBI are ongoing. Seminal work has shown that DTI can provide outcome measures in non-human primates models of traumatic spinal cord injury (Ma et al., 2016) and the remodeling of brain circuits upon hippocampal damage (Meng et al., 2018). Although non-human primate models of TBI has been established (e.g., King et al., 2010), the logistics and the ethics of non-human primates have so far limited the availability for DTI investigations.

Taken together, these findings identify DTI as a key approach to investigate large-scale and microstructural integrity in preclinical models of TBI, with excellent agreement with histological readouts and the advantage of longitudinal, non-invasive assessment.

### DTI Integration With High-Resolution Optical Imaging

There is a growing number of large-scale connectomes for preclinical models obtained with DTI *ex vivo* (Nouls et al., 2018; Allan Johnson et al., 2019) and increasingly *in vivo* (Gimenez et al., 2017; Haber et al., 2017; Müller et al., 2019). At the same time, single-neuron resolution connectomes are being obtained for the whole mouse brain (Ho et al., 2014) or for individual structures (e.g., Commisso et al., 2018) using viral tracing tools and optical imaging. Therefore, the integration of these two techniques appears to be the logical next step. This may take place either in the form of technical approaches enabling the co-acquisition of DTI datasets and MRI-compatible viral tracing or in the form of post-acquisition merging of distinct datasets.

In the domain of *post-hoc* coregistration, the availability of the Allen Brain Connectivity atlas (Ho et al., 2014) has spurred early effort to merge or cross-check DTI atlases with connectivity measures obtained from viral tracing experiments. Initial evaluations in this direction have shown an incomplete match between DTI datasets and tracing datasets, in particular in the connectivity of striatum and cerebellum (Chen et al., 2015). In particular, the DTI connectivity map emerged to be more accurate when the anatomical parcellation was less precise, underscoring the different resolution of the DTI vs. optical imaging. Nevertheless, DTI appears to be reliable in the identification of at least 90% of large projection tracts identified by viral tracing; the misidentified tracts appear to be due to erroneous link of two independent fiber tracts by the tracing algorithm in the DTI dataset (Chen et al., 2015). Therefore, it is anticipated that further improvement of the technology, using machine-learning approaches that can use optical counterparts as “gold standard,” will result in improvement in tracing, in particular in terms of reduced false positives (Maier-Hein et al., 2017). Of note, the resolution of the DTI-MRI datasets for murine models is progressively increasing, thanks to the adoption of ultra-high-field, *ex-vivo* acquisition (Allan Johnson et al., 2019). On the other hand, the acquisition of whole-brain optical images in cleared specimens (Ueda et al., 2020), which are going to have the same overall format of MRI images in terms of explored volume and reference points (and therefore could be normalized to standardized templates), may be anticipated to make for easier and faster integration with DTI-MRI.

Nevertheless, a large degree of complementarity, rather than integration, remains between DTI imaging and optical, tracer-based approaches. In fact, the major advantage for MRI application remains the acquisition of data *in vivo*, which is largely impossible with optical methods; however, DTI datasets are intrinsically devoid of directionality so that the polarization of the identified tracts cannot be deduced solely from diffusion metrics. To this respect, viral tracing provides a critical information, since viruses can be engineered to have anterograde or retrograde propagation (e.g., Commisso et al., 2018).

Of note, DTI atlases are being refined for non-murine animal models of disease. For porcine models, DTI imaging has been used to provide a whole-brain map of connectivity (Simchick et al., 2019) as well as enhanced tractography (Knösche et al., 2015). Furthermore, DTI connectome initiatives have been performed on marmoset (Callithrix jacchus) models (Okano and Mitra, 2015) and in rhesus models (Feng et al., 2017; Young et al., 2017). The correlative histological studies are quickly advancing, especially in marmoset (Goulas et al., 2019; Liu C. et al., 2020; Majka et al., 2020), but also in larger non-human primates (e.g., Decramer et al., 2018 and by the PRIME-DE initiative—PRIMatE Data Exchange (PRIME-DE) Global Collaboration Workshop Consortium, 2020). Thus, the current challenge is to obtain correlative DTI-histological mapping on large brains; the most severe limitations appear to be imposed by the difficulties in optical imaging of large samples with cellular resolution, although promising steps have been already undertaken such as the optical clearing of large organs (Zhao et al., 2020).

## Discussion

### MRI in Preclinical Models of Neurodegeneration

Neuroimaging has provided powerful data on the temporal course of neurobiological changes associated with neurodegenerative disorders and is emerging as a powerful biomarker to define target engagement in therapeutic trials in humans (Masdeu, 2017). Animal studies have a crucial role in neuroscience and have substantially contributed to the understanding of neurodegeneration, and the studies previously presented in the various models of different neurodegenerative disease will pave the way for further DTI-based imaging read-outs in animals, including tracking of changes associated with pharmacological manipulation. Although animal studies are not a substitute for studies in real human biology, animal models provide opportunities for experiments that cannot be performed in humans and thus represent a critical platform upon which translational efforts for treating human neurodegenerative diseases are built (Albanese et al., 2018). Since advanced genetic techniques allow the manipulation of the genome and precise control of gene expression in rodents, transgenic models of human neuropathology are becoming increasingly important. Animal models of brain structure and organization at different neurodegenerative disease stages may define possible read-outs for surrogate markers and enhanced drug trials. Longitudinal studies in animals are of special interest since they can be designed across the entire lifespan of the respective animal in contrast to humans where longitudinal studies have to be designed for many years or even decades to capture a sizeable part of the human lifespan (Gorges et al., 2017). DTI-based analysis of the brain can show disease-related alterations of brain areas, which develop over time. MRI has substantially contributed to the understanding of microstructural brain alterations in animal models in the course of neurodegenerative diseases. Taken together, the broad spectrum of experimental manipulations, which can be longitudinally investigated by the non-invasive DTI approach provides a promising tool for cross-species comparative investigations. The integration of DTI in a multiparametric imaging protocol is a promising approach to integrate microstructural characteristics into a context of the structural and functional networks.

### DTI in Translational Imaging

DTI is a powerful tool providing important information regarding alterations in brain microstructure. As DTI has enormous translational potential, the remaining task is to design animal studies exhausting this potential by focusing on clinically relevant parameters, developmental time points, and by providing carefully matched controls. To assess the axonal damage and myelin degradation, the statistical analysis of DTI metrics can be performed by unbiased whole-brain-based voxelwise comparison or by hypothesis-guided ROI-based or TOI-based quantification. When these tools are combined with the strengths of animal models, a more complete picture of the neurobiological targets and mechanisms of neurodegeneration can be developed. A methodological example of harmonized translational imaging in man and in mouse is a recent cohort DTI study in a mouse model of the Phelan-McDermid syndrome. Here, white matter damage in *SHANK3* deficiency has been investigated in a back-translational study of human subjects with Phelan-McDermid syndrome and terminal deletions of chromosome 22q13, including *SHANK3* on the one hand and a *SHANK3* mouse model on the other hand: with the identical DTI analysis cascade, human DTI data and murine DTI data were analyzed by unbiased voxelwise WBSS-based comparison and by hypothesis-guided TOI-based analysis, respectively (Jesse et al., 2020).

Traditional DTI analysis is using the single-tensor model. While this model can adequately analyze alterations in the microstructure in certain brain regions, it is hardly useful for the representation of crossing fibers, posing substantial problems with robust tractography. A step away from single-tensor representations are high angular resolution diffusion imaging (HARDI) (Ozarslan and Mareci, 2003; Cercignani et al., 2012), Q-ball imaging (Tuch, 2004), and NODDI (Barritt et al., 2018; Gatto et al., 2018d). These approaches rely on acquiring a comparatively high number of diffusion gradient directions to cover a full orientation distribution function. HARDI has been performed in excised tissue (D'Arceuil and de Crespigny, 2007; D'Arceuil et al., 2007), and there have been first attempts to apply HARDI or Q-ball imaging in the whole rodent brain at 16.4 T (Alomair et al., 2015). There are no theoretical restrictions for the application of HARDI or Q-ball imaging to animal cohort studies; however, there are many practical challenges such as scanning time. However, NODDI has been applied to detect neurite orientation dispersion of mouse brain microstructure (Wang et al., 2019) or alterations in hippocampal microstructure (Colon-Perez et al., 2019) and also to investigate spinal cords at 17.6 T in an ALS mouse model (G93A-SOD1 mice) for the detection of presymptomatic axonal degeneration (Gatto et al., 2018d). Because simple Gaussian diffusion models do not sufficiently describe water diffusion in complex tissues, a novel diffusion MRI acquisition approach can be applied, that is, hybrid diffusion imaging (HYDI). HYDI can be fit into NODDI to extract diffusion metrics that may be more biologically specific and unbiased by crossing fibers (Wu et al., 2008, 2018; Daianu et al., 2015a,b). In AD research, both HYDI and NODDI have been applied to rat models (Daianu et al., 2015a) or mouse models (Colgan et al., 2016). Due to their elaborate character, it is unclear if these techniques will be clinically significant, but there are aspects that might aid in the research of neurodegenerative diseases, for example, in the discrimination of cortical gray matter as diffusion MRI is sensitive to architectonic differences between a large number of different cortical areas (Ganepola et al., 2018).

Novel developments show the applicability of deep learning-based techniques to obtain DTI with only six gradients directions (Li et al., 2020). The method uses deep convolutional neural networks to learn the nonlinear relationship between diffusion weighted images and tensor-derived maps, bypassing the conventional tensor fitting procedure, which is well-known to be highly susceptible to noise in diffusion weighted imaging. This technique has been reported and it will be shown in the future if it will contribute to significantly reduce the recording time. The role of DKI (Marrale et al., 2016) has not been generally established yet, although—due to its improved sensitivity to complex microstructural changes—successful studies in TBI (Hansen et al., 2017; Soni et al., 2019) and early microstructural changes in the ALS brain (Gatto et al., 2018b,c, 2019) have already been performed; thus, DKI might be considered to be one of the promising techniques to be included in the data acquisition protocols of future experiments.

### Limitations of DTI

As a macro- or mesoscale imaging technique, DTI has the advantages of 3D and *in-vivo* imaging, including the opportunity of repeated and longitudinal scanning with an arbitrary number of DTI recordings and follow-up scans. The ratio of the microstructural feature size to voxel size is improved in small animal imaging as compared with human or primate imaging; nevertheless, a DTI voxel always contains the averaged information of diffusion covering hundreds of axons as well as the surrounding water molecules. Thus, any DTI metric is a measurement of the physical properties of a volume element without any specific restriction to axonal microstructure. Therefore, DTI metrics determination is only an indirect assessment of fiber density and cell packing, degree of myelination, and individual fiber diameter. Diffusion anisotropy is influenced by axonal integrity or myelin degradation, which are affected by neurodegenerative processes.

There have been many efforts to develop sophisticated techniques to extract relevant information on axonal integrity or myelin degradation to get insights into neurodegenerative processes. This ranges from advanced recording techniques, such as Q-ball, HARDI, or NODDI to extensive postprocessing algorithms, that is, parameterization to one FA value or fiber tracking reconstructions. However, it is an intrinsic property of DTI (which is related to the image recording at a macro- or mesoscale) that any FT technique can only represent the probable fiber bundle course rather than individual axons. As a macro- or mesoscale imaging technique DTI has the advantages of 3D and *in-vivo* imaging, which contains the opportunity of repeated and longitudinal scanning with an arbitrary number of DTI recordings and follow-up scans. The ratio microstructural feature size to voxel size is improved in small animal imaging compared to human or primate imaging; nevertheless, a DTI voxel always contains the averaged information of diffusion covering hundreds of axons as well as the surrounding water molecules. Thus, any DTI metric is a measurement of the physical properties of a volume element without any specific restriction to axonal microstructure. Therefore, DTI metrics determination is only an indirect measurement of density of fibers and cell packing, degree of myelination, and individual fiber diameter. Diffusion anisotropy is influenced by axonal integrity or myelin degradation, which are thought to be mainly affected by neurodegenerative processes.

### Future Aspects

A future aspect of microstructure imaging by DTI (including tractography) is the combination of the detected alteration patterns with further MRI-based techniques such as structural imaging and intrinsic functional connectivity imaging, which can be the basis for connectome imaging as a comprehensive map of neural connections of the species nervous system (Rilling and van den Heuvel, 2018). These techniques have successfully been applied to humans and will further strengthen the translational insights into a complex multiparametric comprehensive structural-functional organization of the animal brains. Such MRI studies can then be expanded, for example, by radioligand neuroimaging or advanced optical imaging (see section DTI Integration With High-Resolution Optical Imaging). There is particular interest in performing these advanced neuroimaging analyses at the group level, given that a trend for bottom-up initiatives is emerging within the neuroscientific community, starting with small-scale projects by single groups that expand upon self-organized collaborations of researchers and infrastructures in “meso-scale” collaborations and develop to grand-scale projects (Kassubek, 2017). Joined forces with a focus on the analysis of specific preclinical models for neurodegeneration might be a solution to the existing challenges especially in preclinical imaging by the combination of neuroimaging data: preferably in a prospectively harmonized design than *ex post facto*, DTI might be one promising advanced imaging candidate in that sense in the light of standardized acquisition protocols with relative robustness. In the context of models of neurodegenerative diseases, both the analysis of disease-related brain changes including the correlation with the clinical phenotype and the analysis of white matter plasticity (Sampaio-Baptista and Johansen-Berg, 2017; Sampaio-Baptista et al., 2020) might facilitate the translational approach of these studies to clinical data.

## Data Availability Statement

The original contributions presented in the study are included in the article/supplementary material, further inquiries can be directed to the corresponding author.

## Author Contributions

H-PM and JK drafted the manuscript. FR and VR revised the manuscript for intellectual content. All authors performed literature investigation, agreed to be accountable for the content of the work, and finally approved the manuscript.

## Conflict of Interest

The authors declare that the research was conducted in the absence of any commercial or financial relationships that could be construed as a potential conflict of interest.
